# Evaluation of food and nutrient intake in a population of subjects affected by periodontal disease with different levels of bone mineral density

**DOI:** 10.3389/fendo.2023.1098366

**Published:** 2023-02-14

**Authors:** Leonardo Guasti, Luisella Cianferotti, Barbara Pampaloni, Francesco Tonelli, Francesco Martelli, Teresa Iantomasi, Maria Luisa Brandi

**Affiliations:** ^1^ Fondazione Italiana Ricerca sulle Malattie dell’Osso (F.I.R.M.O.) Foundation, San Gallo Florence, Italy; ^2^ University of Florence, Piazza di San Marco, Florence, Italy; ^3^ Excellence Dental Network, dell’Ariento, Florence, Italy

**Keywords:** periodontitis, osteoporosis, nutrients intakes, vitamin C, plaque index (PI), food frequency questionnaire (FFQ), food intake

## Abstract

**Introduction:**

Both osteoporosis and periodontitis are pathologies characterized by an imbalance in the bone tissue. Vitamin C is an important factor involved in maintaining the health of the periodontium; its deficiency causes characteristic lesions to periodontal tissues such as bleeding and redness of the gums. Among the essential minerals for the health of the periodontium we find instead calcium.

**Objectives of the study**

The objectives of the proposed study are to study the association between the presence of osteoporosis and periodontal disease. We tried to identify the possible connections between particular dietary patterns and therefore the etiopathogenesis of periodontal disease and secondarily of osteoporosis.

**Materials and methods**

110 subjects were recruited in a single-center observational cross-sectional study carried through the collaboration between the University of Florence and the private institute of dentistry Excellence Dental Network based in Florence, suffering of periodontitis, 71 osteoporotic/osteopenic and 39 non-osteoporotic/osteopenic. Anamnestic data and information on eating habits were collected.

**Results:**

The population showed eating habits that do not meet the intake levels recommended by the L.A.R.N. Regarding the relationship between nutrient intake and plaque index, it appears that in the population, the higher the intake of vitamin C through food, the lower the plaque index value is. This result could reinforce the scientific evidence that there is a protective factor in the onset of periodontal disease by the consumption of vitamin C which to date is still the subject of investigation. In addition, the same type of trend would also have been observed for calcium intake, but a larger sample size would be required to make this effect significant.

**Conclusions:**

The relationship between osteoporosis and periodontitis and the role of nutrition in influencing the evolution of these pathologies still seems to be deeply explored. However, the results obtained seem to consolidate the idea that there is a relationship between these two diseases and that eating habits play an important role in their prevention.

## Introduction

Periodontitis is a chronic inflammatory disease in which bacterial infection of periodontal tissues is necessary and sufficient for the onset and progression of the oral pathology. Yet, numerous other factors negatively affect the course of the disease, like smoking, hormonal changes, endocrine or systemic comorbidities, and poor oral hygiene. It is a condition with poly-microbial etiology which specifically affects the supporting tissues of the teeth. According to the Global Burden of Disease 2010, the global prevalence of severe periodontitis between 1990 and 2010, standardized by age, was 11.2%, i.e. the sixth most widespread disease in the world (1). Age-standardized incidence of the more severe forms in 2010 was similar to that of 1990, with 701 cases per 100,000 individuals per year. Prevalence gradually increased with age, showing a large increase between the third and fourth decades of life, with a peak at about 38 years old.

Osteoporosis is a systemic skeletal disease characterized by a reduction in bone mass and qualitative changes in the material properties of the macro- and micro-architecture of the bones, this leading to an increased risk of fracture even in case of minor traumas (2). Nowadays, densitometry allows to accurately measure the bone mass and BMD (bone mineral density) in g/cm^2^ of bone surface, a property responsible for 60-80% of the mechanical resistance of a bone. According to the WHO (World Health Organization), diagnosis of osteoporosis made by densitometry relies on the assessment of bone mineral density through dual-energy x-ray absorptiometry (DXA) and on its comparison with the average value in healthy adult subjects. Standard deviation from the average peak of bone mass (T-score) is used as unit of measurement. This procedure represents the diagnostic test for osteoporosis and risk of fracture (2). To this regard, it has been observed that risk of fracture starts to exponentially increase with densitometry values ​​of T-score <-2.5 SD, which indeed represents the threshold for diagnosing the presence of osteoporosis.

QUS systems (calcaneus Ultrasonography) are techniques used more and more commonly for densitometry investigations within population groups. Through the measurement of ultrasound variables applied to the calcaneus, they provide a clinical parameter known as Stiffness Index. The Stiffness Index describes the risk of osteoporotic fracture for post-menopausal women comparable to the BMD measured by DXA of the spine or hip.

After reviewing several studies performed using the QUS systems, the FDA approved the use of this procedure for the prevention from the risk of hip fracture, comparable to the DEXA study of the hip/spine.

Based on these diagnostic criteria, approximately 6% of men and 22% of women between 50 and 84 years old in the EU have osteoporosis, i.e. 27.6 million people (3).

Both osteoporosis and periodontitis are characterized by an imbalance between bone tissue resorption and bone tissue neo-genesis, which finally results in bone tissue loss. Furthermore, recent studies suggested the possibility that these two pathologies are inter-connected, playing a role as reciprocal risk factors (4) (5).

Evaluating the possible implications that different diets could have on the oral bacterial population is an aspect still poorly investigated.

A large number of nutrients impact periodontal health, among these macro and micro-elements must be distinguished.

With reference to periodontal disease, the most important macronutrients (carbohydrates, proteins and lipids) are carbohydrates, which are involved in the progressing of periodontal disease associated with dental caries. In fact, high carbohydrate intake seems to promote oral dysbiosis, while its reduction decreases gingival inflammation. Furthermore, diets rich in saturated fats, known to increase oxidative stress, should be avoided in order to prevent the onset of periodontitis (6). To this regard, a higher body fat content has been associated with an increased gingival bleeding in older patients. On the other hand, polyunsaturated fats (such as omega 3) have shown a positive effect on periodontium conditions (7). Other studies conducted instead on protein deprived animals have resulted in the rupture of periodontal ligaments, degeneration of gingival tissues, and resorption of the alveolar bone (8). Another study has finally suggested an inverse relationship between high protein intake and periodontitis (9).

Strong association has also been reported between periodontal disease and obesity (10), a problem that has long been underestimated. Obesity has been indeed identified as risk factor for a number of systemic diseases with inflammatory background. A longitudinal study identified a positive correlation between BMI and the incidence of periodontal disease (6).

The correlation between nutrition and periodontal disease is for various reasons rather uncertain. However, although dental bacterial plaque is accepted as the main causative agent, incorrect dietary can affect both onset and course of the disease.

Several studies have demonstrated the influence of dental plaque as the main etiological factor for gingival inflammation, noting worsening of gingivitis when study participants stopped oral hygiene procedures (11) (12) (13). The authors therefore concluded that the gingivitis experimental protocol is not applicable if the diet does not include refined carbohydrates. Diet seems then to have a strong influence on the gum and on the inflammatory reaction of the periodontal. These effects could affect both tissue repair and immune system mechanisms, which are affected by:

- Decrease in the phagocytic activity of granulocytes;
- Modification of the immune response;
- Alteration in the synthesis of prostaglandins;
- Epithelial changes.

However, although dietary imbalance is not sufficient to induce periodontal disease without the simultaneous presence of the bacterial plaque, it can likewise affect its severity and extension, altering the resistance of the host organism and the regenerative capability of the tissues.

Nutritional recommendations to keep the periodontium in health include reduction of carbohydrate consumption and supplementation of omega 3 fatty acids, vitamin C, vitamin D, antioxidants and fibers (14). *In vitro* studies have shown that vitamin C and D intake may play an important role in the prevention of gingivitis and periodontal inflammation (15).

Evidence in the recent literature reinforces the concept of how important is vitamin D for periodontal health. Periodontal disease seems to be correlated with low vitamin D serum levels; in a cohort of pregnant women over 20 weeks of gestation from the University Hospital “Maggiore della Carità”, Novara, Italy, the authors assessed serum levels of vitamin D and oral health status through the following indexes: Oral Hygiene Index (OHI), Plaque Control Record (PCR), Gingival Bleeding Index (GBI), and Community Periodontal Index of Treatment Needs (CPTIN). They finally found strong correlation between low serum levels of vitamin D and the idexes that identify periodontal disease (16).

Another relevant study indicates that Breast cancer (BC) survivors treated with aromatase inhibitors (AIs) commonly show several pathological issues, including poor oral health, bone health impairment, and vitamin D deficiency. This study evaluates the correlation between oral health and vitamin D status in BC survivors undergoing treatment with AIs through a machine learning approach. It’s showed a significant correlation between DMFT and vitamin D levels; the regression machine learning model showed that vitamin D status and the use of dental floss were the most relevant variables in terms of correlation with Filled Permanent Teeth Index (DMFT). Vitamin D deficiency, inadequate use of dental floss have a negative impact on oral health in BC women (17).

Vitamin C is an important factor for maintaining the health of the periodontium. Its deficiency causes characteristic lesions of the periodontal tissues, such as bleeding and redness of the gums. *In vitro* studies suggest that local applications of vitamin C and magnesium salt decrease inflammation at the level of the gingival fibroblasts (18).

Among the essential minerals for the health of the periodontium there is calcium, which is essential for calcified tissues such as bones and teeth. Deficiencies of this mineral can affect the health of the periodontium. A Danish study demonstrated that a higher dairy intake decreases the severity of periodontitis in adulthood (19).

The aim of this work is therefore to study the association between the presence of osteoporosis and periodontal pathology. We did it by identifying possible connections between specific diets and the etio-pathogenesis of periodontal disease, primarily, and osteoporosis, secondarily.

## Materials and methods

In the scope of a monocentric cross-sectional observational study carried out from November 2019 to December 2021 through the collaboration between the University of Florence and the private dentistry institute Excellence Dental Network based in Florence, were recruited 110 subjects (36 males and 73 females) affected by periodontitis, 71 of which also affected by osteoporosis or osteopenia. Detailed protocol was submitted to and approved by the Ethics Committee.

Criteria for the inclusion in the study were (1) written and signed declaration of informed consent, (2) age ≥ 18 years old, for both sexes, and (3) the diagnosis of periodontitis.

Exclusion criteria were instead (1) to have done antibiotic therapies or (2) steroid therapies in the past 3 months prior to the beginning of the study, (3) presence of parathyroid diseases or (4) diseases related to bone metabolism (except osteoporosis), and (5) development of neoplastic pathologies in the last five years. Subjects presenting eating disorders or pregnancy were also excluded.

Participants, all attending the abovementioned dental institute and all respecting the above listed inclusion criteria, were introduced to the project and provided of the relative documentation (patient information, informed consent, privacy policy). Data was collected anonymously, through the assignment of a specific alphanumeric code to each participant, organized in an electronic database and stored for the purposes expressed in the following scientific research. Two questionnaires for the collection of anamnestic information were administered to the participants, one for information related to the existence of previous pathologies and risk factors for osteoporosis, one for information on eating habits, with particular attention to vitamins and minerals diet intake. An attendance questionnaire already validated by Montomoli and collaborators was also administered (20). Information about potential intake of supplements has also been recorded. The questionnaire consisted of sixteen questions related to nutrient intake. Food selection included in the questionnaire was based on data obtained by the Italian Institute of Nutrition, related to the composition of the Italian diet, to the frequency with which foods are consumed, and their relative importance as sources of calcium. Regarding cheese, more questions were asked to better identify the types of cheese consumed. Main food classes were included in the questionnaire: cereals (pasta, rice, bread and similar and potatoes), fish-meat, eggs, legumes, vegetables, and fruit. Two questions related to the consumption of sweets rich in calcium that are commonly consumed by the Italian population, such as milk-based ice cream and milk chocolate, were also present. Finally, contribution to calcium intake from drinking water was also carefully evaluated, as it can represent an important source of this mineral. A list of the most consumed calcium-rich mineral waters commercialized in Italy has been attached to the Food frequency Questionnaire (FFQ). For each question, participants were also asked to indicate the amount of product consumed, selecting between small, medium, or large portion. In addition to calcium intake, the questionnaire also aimed to estimate the participants’ intake of other macro and micro nutrients important for the health of bones and teeth, i.e. carbohydrates, proteins, lipids, phosphorus, sodium, iron, magnesium, potassium, selenium, zinc, vitamin C, vitamin D, and vitamin B12.

All data collected by the qualified staff of the IRF institute in Microdentistry and the Biomolecular Diagnostic laboratory were transcribed and archived in a database suitably prepared based on the purposes of this study:

- bone mass: those patients who were advised by the dental staff for assessment of the level of bone mineral density performed an ultrasound analysis (QUS) during the same dental visit. Such analysis was used by the dental staff as a primary screening tool to evaluate whether to send the subject for further mineralometric investigations. In case patients were sent for mineralometric analysis (MOC), bone mineral density (BMD) and lumbar and femoral T-score were measured and recorded.
- grading of periodontal disease: the dental staff probed the depth of the periodontal pockets, gingival recession, sub-gingival tartar, pus and bleeding, dental motility, and plaque index. Subjects were classified as suffering from either aggressive or chronic periodontitis (21).

Statistical analysis was carried out using SPSS software and Microsoft Excel. Quantitative data derived from the analysis of parameters relating to osteoporosis, periodontal disease and dietary habits (t-score, bacterial concentrations, frequency of food consumption, etc.) were described through the use of statistical indices of central tendency mean and variability (minimum, maximum, range, standard deviation). For qualitative data, most suitable descriptive statistics (frequency distributions, relative frequencies, etc.) were presented.

After classifying the patients on the basis of the presence or absence of osteoporosis/ostepenia, groups were compared in relation to a series of quantitative variables (bacterial concentrations, daily calcium intake, etc.) using the most suitable statistical tests for independent samples (or an equivalent non-parametric test in the case of non-normal distributions). In addition, the association between osteoporosis/osteopenia and qualitative variables (presence of periodontitis, eating habits) was evaluated.

After having classified patients according to the severity of periodontal disease (mild, moderate, severe), groups were compared in relation to a series of quantitative variables (t-score, blood parameters of bone metabolism) by ANOVA and consequent *post-hoc* corrected with the Bonferroni method (or an equivalent non-parametric test in the case of non-normal distributions). In addition, the association between the severity of periodontal disease and qualitative variables (presence of osteoporosis/osteopenia, eating habits, etc.) was evaluated.

## Results

### Descriptive statistics of the study population

Over the three-year project, 181 individuals were asked to take part in the study, 110 of which agreed to participate. The others refused to participate because not interested.

All 110 participants reported data about the mineralometric and metabolic condition of their bones, genetic analyses and microbiological features of their periodontal.

Only 44 people were finally able to provide anamnestic information and eating habits, this likely because of the unfortunate ongoing pandemic situation, which prevented direct contact with the subjects. Participants were thus contacted by telephone in order to have answers to the questions. Many subjects, at the time of the call, were either not found or no longer interested in participating to the project.

The descriptive data collected are shown below. Overall, we obtained data from 110 subjects, 36 males and 74 females.

### Average age and anthropometry

The average age across the whole sample was 55 years old, while in regard to the anthropometric characteristics the average weight was 67 kg, the average height 1.68 m, and BMI 23.65, i.e. normal weight (**Table 1**).

**Table 1:** Average age and anthropometry.

Age and anthropometry	N	Minimum	Maximum	Average	std. deviation	Asymmetry
	Statistics	Statistics	Statistics	Statistics	Statistics	Statistics
Age	110	27	101	55,22	13,208	,478
Weight (Kg)	107	41,0	120,0	67,250	15,0090	,835
Height (m)	107	1,53	1,89	1,6832	,08513	,271
BMI	107	15,6226	41,9143	23,652578	4,6736212	1,449

### Bone turnover marker and bone mineralometry

Mineralometric data were collected from the entire sample of subjects, obtained through computerized bone mineralometry (MOC) or calcaneus ultrasound (QUS). When possible, data on blood concentration of 25OHD3, PTH, calcium, phosphatemia, alkaline phosphatase, and bone alkaline phosphatase, were also collected (**Table 2**).

**Table 2:** Mineralometric data.

Mineralometric data	N	Average	std. deviation
	Statistics	Statistics	Statistics
Tscore lumbar	15	-2,093	1,5917
Tscore femur tot	13	-1,823	,9671
Tscore femur neck	14	-2,157	,9788
Tscore right foot	99	-,961	1,2538
Tscore left foot	99	-,948	1,2966

On average, the value detected for 25OHD3 is insufficient if compared to the reference values.

The values of PTH, calcium, phosphatemia, alkaline phosphatase and bone alkaline phosphatase are instead in the average when compared to the reference values.

The average t-scores detected in the lumbar and femoral area through MOC examination fall below the desirable values, while the values detected by ultrasonography at the heel are on average in an optimal range, albeit at the limit.

35.5% of the sample had normal mineralometric values, 48.2% had values tending to osteopenia and 16.4% had values testifying a condition of osteoporosis (**Table 3**).

**Table 3:** Mineralometric examination result.

Exam result	Frequency	Valid percentage
normal	39	35,5
osteopenia	53	48,2
osteoporosis	18	16,4
Total	110	100,0

### Grading of periodontal disease

#### Classification, pocket depth (PPD), gingival recession (REC), plaque index

The classification of the severity of periodontal disease was obtained using the classification proposed by Amitage GC (21).

The analysis shows that patients always have generalized forms of periodontitis, in which chronic forms prevail over youthful/aggressive forms; in particular, 16.4% have a mild chronic form, 41.8% a moderate form and 32.7% a severe form (**Table 4**).

**Table 4:** Types of periodontitis found in the population.

Periodontitis (Amitage CG 1999)	Frequency	Valid percentage
Chronic, mild, generalized	18	16,4
Chronic, moderate, generalized	46	41,8
Chronic, severe, generalized	36	32,7
Aggressive, mild, generalized	1	,9
Aggressive, moderate, generalized	1	,9
Aggressive, severe, generalized	8	7,3
Total	110	100,0

The mean pocket depth (PPD) detected by the test was 5.54 mm, therefore beyond the values considered physiological, while the mean gingival recession was 0.59 mm. The average plaque index was found to be 31.16% (**Table 5**).

**Table 5:** Average PPD, REC and plaque index in the population.

	N	Minimum	Maximum	Average	Std.Deviation
	Statistics	Statistics	Statistics	Statistics	Statistics
PPD average (mm)	110	2,20	15,00	5,5414	1,42438
REC average (mm)	110	,00	2,40	,5877	,59648
Plaque Index (%)	97	0	90,48	31,1609	25,10672

### Food questionnaire

#### Consumption of the main foods important for the health of bones and teeth

To the subgroup of people interviewed by telephone was asked about eating habits for estimating calcium intake (20) and other nutrients important for bone and tooth health:

- 38.6% of the sample consumes cow’s milk;
- 47.7% consume yogurt;
- 65.9% consumes hard cheeses;
- 32.6% consume semi-hard cheeses;
- 77.3% consume soft cheeses;
- 20.5% consume ricotta cheese;
- 95.5% consumes pasta/rice;
- 81.8% consume bread;
- 54.5% consume potatoes;
- 93.2% consumes meat/fish;
- 84.1% consume eggs;
- 77.3% consume legumes;
- 95.5% consume vegetables;
- 84.1% consume fresh fruit;
- 29.5% consume ice cream;
- 14.3% consumes milk chocolate;
- 18.2% intake water rich in calcium.

**Tables 6** and **7** show the average weekly intake frequencies and the average portions of the various foods taken into consideration.

**Table 6:** Frequency of consumption of the different food groups.

Weekly consumption frequency			
Foods	N	Average (time/week)	Std. Deviation
Cow’s milk	17	5,94	1,56
Yogurt	21	3,86	2,032
Hard cheeses	29	3,1	1,934
Semi-hard cheeses	14	1,93	1,141
Soft cheeses	34	1,82	1,029
Ricotta cheese	9	1,89	1,054
Pasta/riso	42	4,31	2,054
Bread	36	5,33	2,138
Potatoes	24	1,71	0,751
meat/fish	41	4,2	1,860
eggs	37	2,68	1,547
Legumes	34	2,41	1,598
Vegetables	42	9,29	4,397
Ice cream	13	2,08	1,038
Milk chocolate	6	1,83	1,329

**Table 7:** Average portions of the different food groups.

Medium portion			
Foods/Aliments	N	Avarage (g)	St.deviation
Cow’s milk	17	207,35	59,794
Yogurt	21	129,05	37,504
Hard cheeses	29	64,66	35,955
Semi-hard cheeses	14	62,86	30,237
Soft cheeses	34	91,47	34,455
Ricotta cheese	9	102,78	8,333
Pasta/rice	42	90,95	25,644
Bread	36	95,28	32,38
Potatoes	24	250	106,322
Meat/Fish	41	135,37	30,091
Eggs	37	133,78	77,329
Legumes	34	121,47	47,106
Vegetables	42	176,9	68,876
Ice cream	13	84,62	37,553
Milk chocolate	6	41,67	30,277

In subjects who have declared their intake, milk is consumed almost every day, yogurt more occasionally, cheese only 2-3 times a week on average.

Pasta and bread have an average consumption of 3-4 times a week.

Meat and fish are eaten on average 4 times a week.

Legumes are consumed on average 2 times a week and vegetables at least once a day.

About fruit consumption, the question asked how many fruits per week were consumed; 37 out of 44 subjects replied to consume at least one fruit a week, the rest said they did not consume fruit. The average consumption of fresh fruit in the population was found to be 11.89 fruits per week (standard deviation ± 1479.56), or 1.69 fruits/day.

#### Daily intake of the main nutrients useful for the health of bones and teeth

**Table 8** shows the daily intake values ​​of the main nutrients analyzed, which are important for the prevention of bone and dental health.

**Table 8:** Average intakes of the analyzed nutrients.

	N	Minimum	Maximum	Average	Std. Deviation
	Statistics	Statistics	Statistics	Statistics	Statistics
Ca/die mg	44	218,9843	1507,5456	760,717584	321,6166047
P/die mg	44	385,6909	1482,6531	931,571488	237,3435570
Na/die mg	44	439,9885	1921,2101	973,602366	346,1657990
Fe/die mg	44	2,0775	12,7557	7,893709	2,5206753
Mg/die mg	44	60,8884	254,7129	171,823180	53,2108933
K/die mg	44	550,0149	3386,0009	2105,757209	667,6191528
Se/die mcg	44	11,4049	43,4157	27,981607	8,0220100
Zn/die mg	44	3,6157	14,5086	8,410503	2,2191760
VitC mg	44	4,8416	230,3100	132,337146	60,4172013
VitD mcg	44	,4771	3,1066	1,710894	,7033637
VitB12 mcg	44	1,1937	7,2377	3,843221	1,4569935

Considering the average age of the population (55 years), our data show that the intake of some nutrients in the population included in this study is below the values ​​recommended by the Reference levels of nutrient and energy intake (L.A.R.N.) (22), in particular:

- Calcium;
- iron;
- magnesium;
- potassium;
- selenium;
- zinc;
- vit D;
- vit B12;

result insufficient compared to the recommended daily intake.

### Inferencial statistics- significance

#### Study of the differences in food consumption and nutrient intake between subjects with normal bone density, osteopenic and osteoporotic

By carrying out a univariate ANOVA test and related *post hoc* tests, an attempt was made to investigate whether there are significant differences in the intake of certain foods and nutrients between the different study groups, i.e. subjects with normal, osteopenic and osteoporotic t-score values.

From the analyses shown in **Table 9**, in which only the analyses carried out that have produced significant results are shown, we have obtained:

**Table 9:** ANOVA univariata, study of the differences in food consumption between subjects with normal bone density, osteopenic e osteoporotic subjects.

Dependent Variable	Result	Result	Sig.
Frequency LEGUME	Normal	Osteopenia	0,032
		Osteoporosis	0,332
	Osteopenia	Normal	0,032
		Osteoporosis	0,998
	Osteoporosis	Normal	0,332
		Osteopenia	0,998
Portion VEGETABLES	Normal	Osteopenia	0,01
		Osteoporosis	0,038
	Osteopenia	Normal	0,01
		Osteoporosis	0,852
	Osteoporosis	Normal	0,038
		Osteopenia	0,852
Frequency VEGETABLES	Normal	Osteopenia	0,559
		Osteoporosis	0,122
	Osteopenia	Normal	0,559
		Osteoporosis	0,379
	Osteoporosis	Normal	0,122
		Osteopenia	0,379

• Significant difference in the portion and frequency of consumption of fresh vegetables between normal and osteopenic subjects (higher consumption);

• Significant difference in the portion and frequency of vegetable consumption between normal and osteoporotic subjects (higher consumption);

• Significant difference in the frequency of consumption of legumes between normal and osteopenic subjects (higher consumption);

• Significant difference in the frequency of consumption of legumes between normal and osteoporotic subjects (higher consumption). The other analyzes did not give significant results.

As regards the intake of the different nutrients in the three different categories of subjects, the following was found:

- Significant difference in Fe intake between normal and osteopenic subjects (higher intake);
- Significant difference in Mg intake between normal subjects and osteopenic (higher) intake;
- Significant difference in K intake between normal and osteopenic subjects (higher intake);
- Significant difference in vitamin C intake between normal and osteopenic subjects (higher intake);
- Significant difference in vitamin C intake between normal and osteoporotic subjects (higher intake);

The p values are shown in **Table 10**.

**Table 10:** ANOVA univariata study of the differences in food consumption between subjects with normal bone density, osteopenic e osteoporotic subjects.

Dependent variable	Result mineralometry	Result mineralometry	Error std.	Sig.
Ca/die_mg	Normal	Osteopenia	103,5798	0,847
		Osteoporosis	161,6607	0,477
	Osteopenia	Normal	103,5798	0,847
		Osteoporosis	162,5093	0,697
	Osteoporosis	Normal	161,6607	0,477
		Osteopenia	162,5093	0,697
P/die_mg	Normal	Osteopenia	76,30852	0,436
		Osteoporosis	119,0975	0,914
	Osteopenia	Normal	76,30852	0,436
		Osteoporosis	119,7227	0,921
	Osteoporosis	Normal	119,0975	0,914
		Osteopenia	119,7227	0,921
Na/die_mg	Normal	Osteopenia	111,4268	0,472
		Osteoporosis	173,9078	0,824
	Osteopenia	Normal	111,4268	0,472
		Osteoporosis	174,8207	0,986
	Osteoporosis	Normal	173,9078	0,824
		Osteopenia	174,8207	0,986
Fe/die_mg	Normal	Osteopenia	0,717718	0,004
		Osteoporosis	1,120168	0,233
	Osteopenia	Normal	0,717718	0,004
		Osteoporosis	1,126048	0,849
	Osteoporosis	Normal	1,120168	0,233
		Osteopenia	1,126048	0,849
Mg/die_mg	Normal	Osteopenia	15,55401	0,013
		Osteoporosis	24,27571	0,167
	Osteopenia	Normal	15,55401	0,013
		Osteoporosis	24,40314	0,999
	Osteoporosis	Normal	24,27571	0,167
		Osteopenia	24,40314	0,999
K/die_mg	Normal	Osteopenia	197,1462	0,015
	Normal	Osteoporosis	307,6932	0,381
	Osteopenia	Normal	197,1462	0,015
		Osteoporosis	309,3084	0,858
	Osteoporosis	Normal	307,6932	0,381
		Osteopenia	309,3084	0,858
Se/die_mcg	Normal	Osteopenia	2,515653	0,158
		Osteoporosis	3,92627	0,756
	Osteopenia	Normal	2,515653	0,158
		Osteoporosis	3,946881	0,881
	Osteoporosis	Normal	3,92627	0,756
		Osteopenia	3,946881	0,881
Zn/die_mg	Normal	Osteopenia	0,69102	0,178
		Osteoporosis	1,0785	0,333
	Osteopenia	Normal	0,69102	0,178
		Osteoporosis	1,084161	0,96
	Osteoporosis	Normal	1,0785	0,333
		Osteopenia	1,084161	0,96
VitC_mg	Normal	Osteopenia	17,35689	0,013
		Osteoporosis	27,08952	0,046
	Osteopenia	Normal	17,35689	0,013
		Osteoporosis	27,23173	0,844
	Osteoporosis	Normal	27,08952	0,046
		Osteopenia	27,23173	0,844
VitD_mcg	Normal	Osteopenia	0,225023	0,5
		Osteoporosis	0,351201	0,912
	Osteopenia	Normal	0,225023	0,5
		Osteoporosis	0,353045	0,502
	Osteoporosis	Normal	0,351201	0,912
		Osteopenia	0,353045	0,502
VitB12_mcg	Normal	Osteopenia	0,472399	0,81
		Osteoporosis	0,737289	0,88
	Osteopenia	Normal	0,472399	0,81
		Osteoporosis	0,74116	0,659
	Osteoporosis	Normal	0,737289	0,88
		Osteopenia	0,74116	0,659

The other analyses did not give significant results.

#### Evaluation of the correlations between the plaque index and nutrient intake (calcium, carbohydrates and vitamin C)

Using the Spearman rank correlation coefficient, it was investigated whether there was a correlation between the intake values of certain nutrients important for bone and tooth health (calcium, carbohydrates and vitamin C) and the plaque index values (**Table 11**):

- as the vitamin C intake value decreases, the plaque index value in the entire population increases;
- for Calcium and Carbohydrates, no correlation is observed for too low a sample size.

**Table 11:** Spearman’s correlation, evaluation of the correlations between the plaque index and nutrient intake (calcium, carbohydrates and Vit C).

			Plaque Index	
Rho di Spearman	Plaque Index	Correlation coefficient Sig. (2-code) N	1,000	
	
			97	
	VitC_mg	Correlation coefficient Sig. (2-code) N	-,385	
			,025	
			34	
	Ca/die_mg	Correlation coefficient Sig. (2-code) N	-,199	
			,259	
			34	

## Discussion

### Descriptive evaluations

The data collected show us that the population involved in this study is made up of a group of subjects tending to belong to the over 50 age group, with BMI values within the recommended range, for both the male and female population. With regard to the subgroup to which it was possible to administer the anamnestic questionnaire, a population that tends to be “healthy” emerged. The data relating to bone turnover and mineralometric values describe a population with blood values of PTH, calcemia and phosphatemia tendentially in line with the desirable values, but with values of 25OHD3 below the optimal value. Furthermore, the t-score values of both QUS and MOC, when available, identify a population mainly made up of fragile subjects from the point of view of the risk of fracture and osteoporosis, with a smaller number of subjects with values tending to normal, which they were then compared for the variables under study with the more frail subjects.

### Periodontal and bone evaluations

The assumption on which subjects were recruited for participation to the study was that of being affected by periodontal disease. On this regard, sample analysis shows that the most represented forms have a chronic rather than an aggressive nature, especially of moderate or advanced type, with high gingival recession values and periodontal pocket depths in the tested sites.

### Nutritional evaluations

The population that provided answer to the food questionnaire showed eating habits that do not meet the intake levels recommended by the L.A.R.N. Nutrients important for bone health and relevant for the prevention of periodontal disease and mouth health in general, such as calcium, iron, magnesium, potassium and fiber, were found to be below the recommended values. It is no coincidence that these nutrients are mainly taken from foods of plant origin, such as fruit, vegetables and legumes. Foods that have proved to be deficient in the consumption habits of the population, both in terms of frequency of consumption and in amount consumed per portion. About the calcium intake, it is interesting to note that in our population this nutrient mainly derives from the intake of cheese, which also bring nutrients that are dangerous for health when consumed in excess (cholesterol and triglycerides), rather than from leaner foods, (milk and yogurt) which seem to be consumed much less frequently on average. This situation brings the attention on the need for a more efficient dietary education of the patients. To this regard, the dental staff could play an important intermediary role between patients and nutrition specialists, like nutritionists and dieticians, in order to limit and prevent nutritional imbalances predisposing to a whole series of pathologies like the periodontitis itself, osteoporosis, dismetabolisms and hypertension. Quite relevant results emerged from the analysis of significance and correlation; Specifically, it seems that subjects with lower t-score values, and therefore predisposed to a greater risk of bone fragility, show unsatisfactory but still higher intakes on average for many nutrients important for bone health among those that we have already mentioned (Fe, Mg, K, vit C, fibers). This result could be explained by the fact that a subject already defined at risk of developing pathology has already been sensitized, at least in part, to the importance of nutrition in the prevention of these pathologies by their generic physician or likewise by health personnel previously encountered. Real primary prevention, the one intended for healthy subjects, would therefore be an element of greater criticality, as people who still do not perceive a problem would tend to be less attentive to their eating habits. For this reason, it would be essential in the future to increase awareness of food choices even in healthy subjects, in order to prevent any nutrient shortcomings and future pathological developments.

Another result of great interest was obtained about the relationship between nutrient intake and plaque index, a factor that describes the severity of periodontal disease. From the correlation analyzes carried out, it appears that across the population, the higher the intake of vitamin C through food, the lower the plaque index value is. This result could reinforce the scientific evidence that there is a protective factor in the onset of periodontal disease from the consumption of vitamin C, which is currently subject of investigation and debate). Systematic reviews on this aspect were performed in latest years; in 2019 Akio et al. selected 14 articles corresponding to inclusion criteria after a full revision of 716 articles. The vitamin C intake and blood levels were negatively related to periodontal disease in all seven cross-sectional studies. The subjects who suffer from periodontitis presented a lower vitamin C intake and lower blood-vitamin C levels than the subjects without periodontal disease in the two case-control studies. The patients with a lower dietary intake or lower blood level of vitamin C showed a greater progression of periodontal disease than the controls. The intervention using vitamin C administration improved gingival bleeding in gingivitis, but not in periodontitis. Alveolar bone absorption was also not improved. The present systematic review suggested that vitamin C contributes to a reduced risk of periodontal disease (23). In 2021 Hytham N. et al. performed another systematic review in which they found six studies fulfilled the inclusion criteria. Vitamin C supplementation helped improve bleeding indices in gingivitis but did not significantly lead to reduction of probing depths or clinical attachment gain for periodontitis. In this case, administration of vitamin C as an adjunct to non-surgical periodontal therapy did not result in clinically significant improvements in pocket probing depths at 3 months in periodontitis patients. With the limited evidence available, no recommendation can be made for supplementation of vitamin C in conjunction with initial periodontal therapy for subjects with periodontitis to improve primary treatment outcome measures. (24).

Once this aspect will be consolidated, we could then focus on the role that this vitamin plays in the prevention of the osteoporotic disease (25), another aspect currently under investigation, and define its importance in the prevention from the two diseases. Furthermore, a similar trend would have also been observed for calcium intake, but a larger sample size would be required to make this effect significant. In the future, we hope to be able to deepen the relationship of this nutrient with the prevention of periodontal disease, which is already fundamental in the prevention of bone health.

### Conclusions

The relationship between osteoporosis and periodontitis and the role of nutrition in influencing the course of these pathologies seems still to be extensively explored. However, our results consolidate the idea that there is a relationship between these two diseases, and that eating habits play an important role in their prevention. The analyses presented here may be of great interest for the development of future studies aiming to expand the sample size and to reduce the confounding factors present at the level of the studied populations.

## Data availability statement

The original contributions presented in the study are included in the article/supplementary material. Further inquiries can be directed to the corresponding authors.

## Ethics statement

The studies involving human participants were reviewed and approved by Comitato etico regionale per la sperimentazione clinica della regione Toscana. The patients/participants provided their written informed consent to participate in this study.

## Author contributions

LG: Main author and performer of the study. LC: Supervisor of the project. BP: Revisor of the main article. FT: organized the collaboration and the cooperation between F.I.R.M.O. and EDN. FM: Main Chief of EDN. TI: organized the collaboration and the cooperation between F.I.R.M.O. and University of Florence. MB: Main Chief of F.I.R.M.O. All authors contributed to the article and approved the submitted version.
